# Exploring treatment adherence in long-term sick-listed workers and the impact of coping strategies, illness perceptions and perceived health

**DOI:** 10.1186/s12889-022-12676-1

**Published:** 2022-02-08

**Authors:** Tialda Hoekstra, Loes Wilming, Christiaan Sjobbema, Sandra Brouwer

**Affiliations:** 1 Department of Health Sciences, Community and Occupational Medicine, University Medical Center Groningen, University of Groningen, PO Box 196, 9700 AD Groningen, the Netherlands; 2 Research Center for Insurance Medicine, Amsterdam, the Netherlands; 3 The Dutch Social Security Institute: the Institute for Employee Benefits Schemes (UWV), Amsterdam, the Netherlands

**Keywords:** Occupational health, Return-to-work, Sick leave, Patient adherence, Disability benefit, Chronic disease

## Abstract

**Background:**

Treatment adherence is important to improve return to work in sick-listed workers. Especially in long-term sick-listed workers who apply for a disability benefit and therefore have not (fully) returned to work, it is of great value to gain insight in the adherence to advice of physicians. Non-adherence could be one of the main reasons why they have not returned to work and are sick-listed for a long-term. The aim of the study is to explore treatment adherence and possible associated factors to advice from medical and occupational health physicians in long-term sick-listed workers.

**Methods:**

The study is a cross-sectional survey study among 561 long-term (partly) sick-listed workers applying for a disability benefit. Associations of sociodemographic characteristics, disease related factors, coping strategies (Utrecht Coping List, UCL), illness perception (Illness Cognition Questionnaire, ICQ) and perceived health (Short-Form 12, SF12) with treatment adherence (measured with the Medical Outcomes Study Measures of Patient Adherence, MOS-MPA) were analysed separately for adherence to medical advice (*n* = 348, mean age 51.3 ± 9.1 years, 55.9% female) and adherence to occupational advice (*n* = 229, mean age 50.4 ± 9.5 years, 54.1% female).

**Results:**

Among participants, 63.3% to 76.4% reported they were able to do what the physician told them to do. However, about half of the participants found it easy to follow-up and implement the suggestions of the physician (54.3% for medical advice and 50.2% for occupational advice). Having a mental health disorder was negatively associated with adherence to medical advice. An active coping strategy, acceptance of the disease, and perceiving positive long-term consequences of the disease were associated with a higher adherence, whereas focusing on the negative consequences was associated with a lower adherence, both for medical and occupational advice.

**Conclusions:**

The tendency to adhere to medical and occupational advice in long-term sick-listed workers is relatively low. In order to increase return to work in this population, medical and occupational health physicians should especially be aware of the adherence of sick-listed workers with mental health disorders, but also on those who focus on the negative consequences of their (physical or mental health) disorder.

## Introduction

Having a job is fundamental to social inclusion, but employment opportunities for people with disabilities are limited. Employment rates of people with disabilities are 35% lower compared to people without disabilities, and are associated with work loss [1, 2]. Around 6% of the working-age population rely on disability benefits [1]. Besides the economic impact for society, work disability may have huge impact for the workers themselves and their families [3]. People with disabilities are often faced with additional difficulties such as relational, domestic, addiction, financial or educational problems [1, 4]. The longer workers are sick-listed and remain out of the workplace, the less likely they are to return to work (RTW), even if they are still motivated to RTW [5].

To prevent sick listed workers from receiving long-term disability benefits, several programs and interventions have been developed to increase RTW. However, a review by Vogel and colleagues [6] on the effect of 14 randomized controlled trials to improve RTW for workers on sick leave for at least four weeks, showed that both for short-term and long-term follow-up, none of the interventions were effective [6]. Compared to usual practice, there were no significant differences among outcomes; time to RTW, cumulative sickness absence, the proportion of participants working at follow-up, or the proportion of participants who returned to work. These results might suggest that other factors, independent of the intervention, play a role in the aim to increase RTW and prevent early labour market exit due to unemployment, disability benefits, and early retirement.

Clinical studies suggest one of the most important aspects of treatment success is patient adherence to treatment. Previous studies have shown that non-adherence to treatment is very common, reaching up to 70% in chronic diseases [7, 8]. Patient-related factors, such as younger age, male gender and lower educational level, and psychological factors, such as poor insight and denial of the illness, are risk factors for medication non-adherence [8]; further, non-adherence has been found to be associated with negative treatment outcomes. Furthermore, medication adherent workers with a chronic disease are fewer days absent from work and on shorter-term disability than non-adherent workers [9].

Literature is available on medical treatment adherence, but treatment adherence in the RTW process to occupational advice is lacking. There is no knowledge if long-term sick-listed workers adhere to medical and occupational advice given by medical specialists and occupational health physicians, or on factors related to adherence in this population. Especially in long-term sick-listed workers who apply for a disability benefit and therefore have not (fully) returned to work, it is of great value to gain insight in the adherence to advice of physicians. Non-adherence could be one of the main reasons why they have not returned to work and are sick-listed for a long-term. Additionally, there is a lack of evidence which personal factors are associated with treatment adherence of long-term sick-listed workers. Future RTW interventions might then focus on these factors first, to increase treatment adherence, which may subsequently lead to an increase in returning to work.

The aim of the present study is to 1) explore adherence to treatment advice provided by medical and occupational health physicians, 2) study associations between sociodemographic characteristics and disease related factors, as determinants, and medical and occupational treatment adherence, as outcomes, and 3) study associations between coping strategies, illness perceptions and perceived health, as determinants, and medical and occupational treatment adherence, as outcomes, in long-term sick-listed workers applying for disability benefits.

## Materials and methods

### Study design, procedure and study population

The study included data from a cross-sectional survey study among long-term sick-listed employed workers (18–65 years) applying for a disability benefit at The Dutch Social Security Institute: the Institute for Employee Benefits Schemes (UWV). The UWV is responsible for all work disability claim assessments under social security regulations. To be eligible for disability benefits in the Netherlands one has to be sick-listed for 88 weeks and not fully returned to work. Individuals may receive disability benefits for a disease or handicap due to either occupational or non-occupational causes.

All applicants for a disability benefit, being sick-listed for 88 weeks and not fully recovered, were found eligible for the study. Applicants could therefore be fully sick-listed and not working at all or being partly sick-listed and still able to work but not for the complete number of hours of their contract. Participants were recruited using registry data from eight UWV offices across the Netherlands (Den Haag, Leiden, Den Bosch, Tilburg, Eindhoven, Leeuwarden, Assen, and Emmen). Applicants received an information letter, an invitation letter to participate in the study, an informed consent form and a prepaid envelope. If participants agreed, they were asked to return the signed consent form in the prepaid envelope within two weeks. After the signed consent form was received by the researcher, the applicant was sent a paper version of the survey at their home address. The survey took 20–30 min to complete and had to be returned to the researcher in a prepaid envelope within two weeks. Participation was voluntary and telephone support was continuously available during the recruitment period.

Data collection started in June 2015 and ended in March 2016. In total 5,407 work disability benefit applicants were contacted about the study. Of these, 4241 (78.4%) did not respond, 511 (9.5%) refused to participate, and 655 (12.1%) agreed to participate and received the questionnaire. Of the 655 disability benefit applicants who agreed to participate, 561 (85.6%) applicants returned a completed questionnaire and were included in the study. For the current study on adherence, only participants who reported having contact with a physician in the past three months, having received advice and completed the adherence questionnaire were included.

Written consent was provided by all study participants. Ethical approval was sought from the Medical Ethics Committee of the University Medical Center Groningen, which advised that, according to Dutch law, ethical clearance was not required for this survey study. The study was performed in accordance with the principles outlined in the Declaration of Helsinki.

### Measurements

The study was set up within the framework of Bandura’s Social Cognitive Theory, adjusted to a setting of the return to work process after long-term sickness absence of persons eligible for disability benefit assessment [10]. Personal and behavioural measurements were selected guided by this theory, and the ability to change.

#### Treatment adherence

Treatment adherence was assessed with the Dutch version of the Medical Outcomes Study Measures of Patient Adherence (MOS-MPA) questionnaire [11, 12]. The five-item questionnaire measures the tendency of participants to adhere to medical advice given by the physician. Participants were first asked if, during the past three months, they had contact with their general practitioner or a medical specialist. Second, if they had received advice from the general practitioner or the medical specialist. If they gave a confirmative answer to both questions, the MOS-MPA questionnaire was offered to fill out regarding the adherence to the medical advice. The same procedure was followed with regards to contact with and advice from an insurance or occupational physician.

The questionnaire included the following items: How often during the past three months applied the following statements to you? (1)”I had a hard time doing what the physician suggested I do”; (2) "I followed my physician's suggestions exactly”; (3) "I was unable to do what was necessary to follow my physician's treatment plans"; (4) "I found it easy to do the things my physician suggested I do"; and (5) "Generally speaking, how often during the past three months, were you able to do what the physician advised you to do?". Answers were given on a six-point scale ranging from 1 = none of the time, to 6 = all of the time. To score the general adherence scale first items 1 and 3 were reversed. Second, the responses were averaged and then transformed to a 0 to 100 linearly distribution. A higher score indicated better adherence.

Cronbach’s alpha of the adherence to medical treatment advice given by the general practitioner or medical specialist and the occupational advice given by the insurance or occupational physician were 0.80 and 0.83 respectively.

#### Sociodemographic characteristics and disease related factors

Age, gender, educational level, work status, type and number of chronic disease(s) were included in the questionnaire. The highest education level successfully completed was categorized into low (no education, primary, lower vocational and lower secondary education), medium (intermediate vocational and high school) and high (higher vocational and university).

Work status was operationalized as not working (zero hours per week) or working (> 1 h per week).

The presence of a chronic disease was based on the open question: ‘Which diagnosed medical condition(s) is/are causing work disability? The answers were classified to International Classification of Diseases 10^th^ edition (ICD-10) categories by two independent insurance physicians. In case of disagreement, the case was discussed until consensus was reached. The main five diagnoses (musculoskeletal disease, mental health disorder, neoplasm, disease of the nervous system and cardiovascular disease) are categorized in this study, all other diagnoses were categorized as ‘other disease’.

Multimorbidity was defined by having two or more chronic diseases, based on the number of diseases reported by the participant.

#### Coping strategies

Coping strategies were measured with the Utrecht Coping List (UCL) [13]. The UCL is a questionnaire with seven subscales that represent different coping styles. In the current study, two subscales were selected: active approach (seven items) and avoidance/abide (eight items), resulting in a 15-item questionnaire. All items relate to the way a person acts to minimize the impact of stressful events and are answered on a four-point scale, with 1 = seldom or never to 4 = very often. Item scores related to the subscales were summed to create a total score of each subscale, resulting in theoretical scoring ranges of 7–28 for active approach and 8–32 for avoidance/abide, higher scores indicate a greater tendency to adopt that particular coping strategy. Cronbach’s alpha of the subscales in the current study were 0.86 for active approach and 0.73 for avoidance/abide, indicating a good internal reliability.

#### Illness perceptions

Illness perceptions were assessed with the Illness Cognition Questionnaire (ICQ), which measures generic illness beliefs across chronic conditions [14]. The ICQ is a 18-item questionnaire that contains three six-item scales related to cognitive ways patients ascribe meaning to chronic illness: helplessness (focusing on the negative consequences of the disease and generalizing them to functioning in daily life; e.g.: “My illness limits me in everything that is important to me”), acceptance (acknowledging being chronically ill and perceiving the ability to manage the negative consequences of the disease; e.g.: “I have learned to live with my illness”) and perceived benefits (also perceiving positive, long-term consequences of the disease, e.g.: “Dealing with my illness has made me a stronger person”). Items are scored on a four-point scale (1 = not at all, 2 = somewhat, 3 = to a large extent, 4 = completely). Scale scores for the three illness perceptions are calculated by summing up the item scores, resulting in theoretical scoring ranges of 6–24 for each subscale. Higher scores indicate that the illness perception is stronger present in the participant. The ICQ has strong internal consistency and reliability, and good construct and predictive validity across chronic conditions [14, 15]. Cronbach's alpha in the current study was 0.62 for helplessness 0.90 for acceptance, and 0.86 for perceived benefits.

#### Perceived health

Perceived health was measured with the 12-item Short-Form Health Survey (SF-12), a practical, reliable and valid measure of physical and mental health [16]. The SF-12 is a brief inventory of self-reported mental and physical health. SF-12 scores were recoded, standardized to a 0–100 scale and summarized using a syntax included in the SF-12 manual into two summary scores, i.e. Physical Health Composite Scores (PCS) and Mental Health Composite Scores (MCS). Higher scores indicate a better health.

### Statistical analyses

Analyses were performed separately for adherence to medical advice and adherence to occupational advice.

Descriptive statistics (frequencies, percentages, means and standard deviations) were used to characterize the study population and to gain more insight in the tendency to adhere to advice given by medical and occupational health physicians.

To examine the associations of age, gender, educational level, work status, ICD-10 diagnosis, and multimorbidity with treatment adherence, univariable and multivariable linear regression analyses were conducted. First, all variables were, independently, analysed using univariable linear regression analyses, to study the unadjusted association with treatment adherence of each variable. Second, all variables with a p-value < 0.20 in the univariable analyses were included in the multivariable linear regression analysis, to explore the independent associations with treatment adherence. A p-value of 0.20 was used for the selection of the variables in the multivariable analyses, as a stricter p-value can fail in identifying variables known to be important [17].

To examine the associations of coping strategies, illness perceptions and perceived health with treatment adherence, univariable and multivariable linear regression analyses were conducted for each questionnaire and/or subscale separately, adjusting for age, gender, educational level, work status, multimorbidity and ICD-10 diagnosis in the multivariable analyses.

Additionally, sensitivity analyses were performed to gain more insight in the characteristics of participants who adhere to both medical and occupational treatment advice. For this specific analysis, participants were included who reported having contact with both an occupational physician and a medical specialist, having received advice from both physicians, and completed both adherence questionnaires. Four groups were created: 1. both low adherence, 2. high medical adherence + low occupational adherence, 3. high occupational adherence + low medical adherence, and 4. both high adherence, based on the question ‘Generally speaking, how often during the past three months, were you able to do what the physician advised you to do?’ of the adherence questionnaire. Low adherence was defined by answering the question with ‘none of the time’, ‘almost never’ or ‘sometimes’. High adherence was defined when the question was answered with ‘often’, ‘almost always’ or ‘all of the time’. Chi-square tests and ANOVA were used to examine significant differences of these four groups on gender, age, educational level, ICD-10 diagnosis, multimorbidity and work status.

All analyses were carried out using the IBM Statistical Package SPSS version 26. A two-tailed p-level less than 0.05 was considered to indicate statistical significance.

## Results

### Study sample

**Fig. 1:**
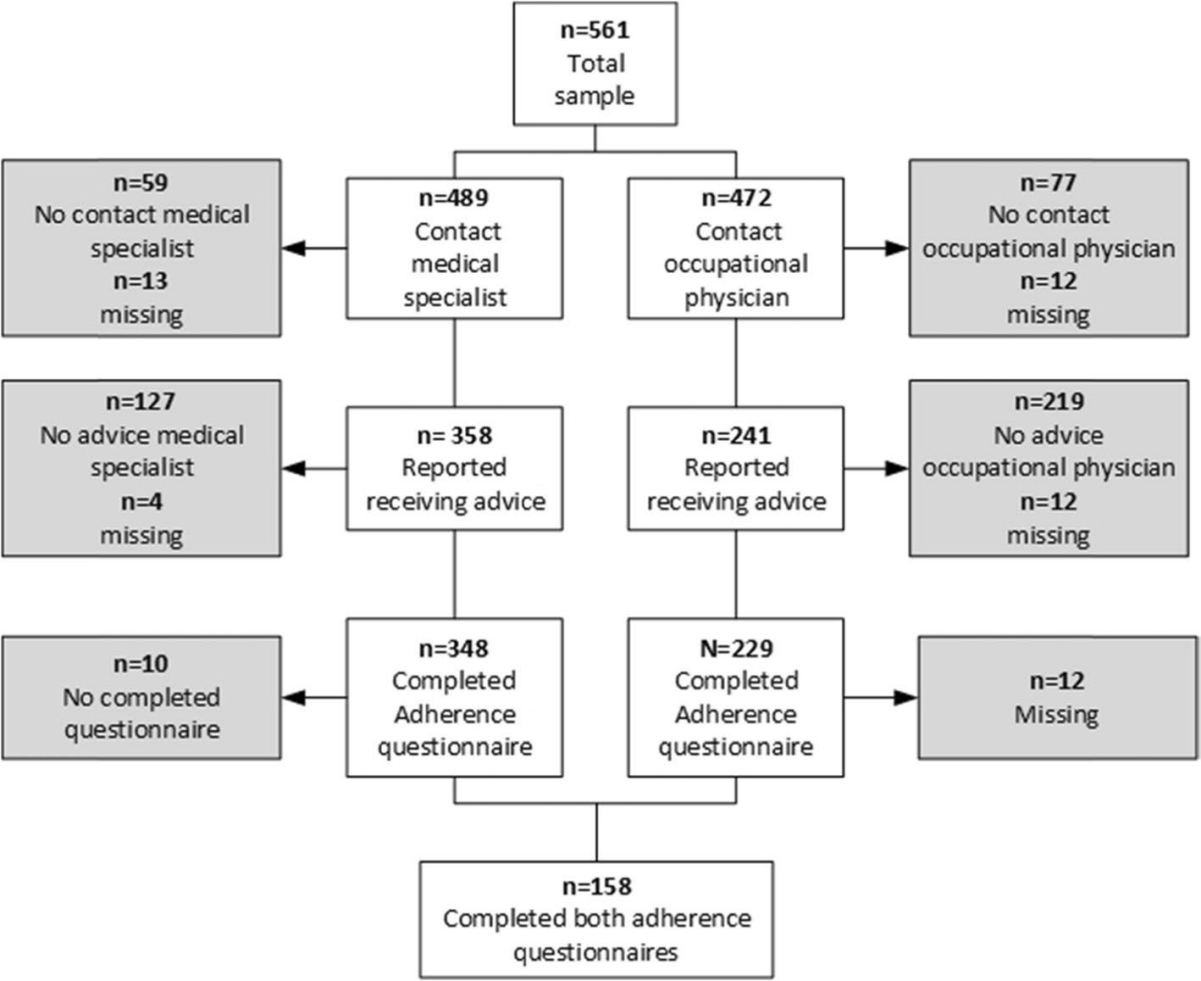
Flowchart of the study sample

Of the 561 study participants, 348 (62.0%) participants reported they had contact with a medical specialist during the past three months, received medical advice and completed the adherence questionnaire (Fig. 1). Of these participants the mean age was 51.3 ± 9.1 years, 55.9% were female, 42.9% had a low educational level, and 36.2% was working (Table 1). The study sample on medical adherence (*n* = 348) differed significantly on having a disease of the nervous system (15.5% versus 8.3%) and multimorbidity (48.6% versus 38.7%) from the excluded participants; those who did not have contact with a medical specialist, received medical advice and/or completed the adherence questionnaire(*n* = 213).

**Table 1:** Characteristics of the study population

	Medical treatment adherence (*n* = 348) N^a^ (%) or mean (± SD)	Occupational treatment adherence (*n* = 229) N^a^ (%) or mean (± SD)
Age in years	51.3 (± 9.1)	50.4 (± 9.5)
Gender		
Male	152 (44.1%)	105 (45.9%)
Female	193 (55.9%)	124 (54.1%)
Educational level		
Low	149 (42.9%)	76 (33.2%)
Medium	123 (35.4%)	91 (39.7%)
High	75 (21.6%)	62 (27.1%)
Working	126 (36.2%)	121 (52.8%)
Diagnosis		
Musculoskeletal disease	148 (42.5%)	74 (32.3%)
Mental health disorder	96 (27.6%)	57 (24.9%)
Neoplasm	54 (15.5%)	35 (15.3%)
Disease of the nervous system	54 (15.5%)	29 (12.7%)
Cardiovascular disease	54 (15.5%)	36 (15.7%)
Multimorbidity	169 (48.6%)	87 (38.0%)
Coping strategies^**b**^		
Active approach	18.2 (± 4.3)	19.1 (± 4.0)
Avoidance / abide	16.7 (± 3.9)	16.3 (± 3.6)
Illness perceptions^**c**^		
Helplessness	16.2 (± 4.1)	15.0 (± 4.1)
Acceptance	12.8 (± 4.2)	13.6 (± 4.1)
Perceived benefits	12.9 (± 4.3)	13.6 (± 4.3)
Perceived health^**d**^		
Physical health	29.8 (± 9.5)	32.5 (± 10.4)
Mental health	42.7 (± 7.2)	42.8 (± 6.8)

*N* number of participants, *SD* standard deviation

^a^Numbers do not always add up to sample n due to missing data

^b^Coping strategies measured with the Utrecht Coping List (UCL) [13]

^c^Illness perceptions measured with the Illness Cognition Questionnaire (ICQ) [14, 15]

^d^Perceived health measured with the Short-Form 12 (SF-12) [16]

Of the total study sample, 229 (40.8%) participants reported having contact with an occupational physician, received occupational advice and completed the questionnaire (Fig. 1). These participants had a mean age of 50.4 ± 9.5 years, 54.1% were female, 33.2% had a low educational level, and 52.8% were working (Table 1). Study participants on occupational adherence (*n* = 229) were more educated (33.2% versus 51.2%), had lower musculoskeletal system disease prevalence (32.3% versus 52.6%) and less multimorbidity (38.0% versus 49.8%), and were more often working (52.8% versus 26.1%) than excluded participants (*n* = 332) within the total study sample.

### Treatment adherence

The mean score for the tendency to adhere to advice given by medical specialists was 69.8 ± 19.4, and for advice given by occupational health physicians 67.0 ± 22.1 (Table 2). About 80% of the participants indicated they did not have a hard time doing what the physician said (81.0% for medical advice and 78.2% for occupational advice) and were able to do what was necessary to follow the advice (83.0% and 80.3% respectively). However, about half of the participants found it easy to do the things the physician suggested them to do (54.3% for medical advice and 50.2% for occupational advice). Participants who received advice from the medical specialists were in general more often able to do what the physician told them to do compared to participants who received advice from the occupational health physicians (respectively 76.4% and 63.3%) (Table 2).

**Table 2:** Treatment adherence measured with the MOS-MPA questionnaire separately for medical and occupational adherence

	Medical treatment adherence (*n* = 348)		Occupational treatment adherence (n = 229)	
	None of the time, almost never, sometimes N ^a^ (%)	All of the time, almost always, often N ^a^ (%)	None of the time, almost never, sometimes N ^a^ (%)	All of the time, almost always, often N ^a^ (%)
I had a hard time doing what the physician suggested I do	282 (81.0%)	66 (19.0%)	179 (78.2%)	50 (21.8%)
I followed my physician’s suggestions exactly	37(10.6%)	311 (89.4%)	40 (17.5%)	189 (82.5%)
I was unable to do what was necessary to follow my physician’s treatment plans	298 (83.0%)	59 (17.0%)	184 (80.3%)	45 (19.7%)
I found it easy to do the things my physician suggested I do	159 (45.7%)	189 (54.3%)	114 (49.8%)	115 (50.2%)
Generally speaking, how often during the past three months, were you able to do what the physician told you?	82 (23.6%)	266 (76.4%)	84 (36.7%)	145 (63.3%)
General treatment adherence score (mean, ± SD)	69.8 (± 19.4)		67.0 (± 22.1)	

*N* number of participants, *SD* standard deviation

^a^ Numbers do not always add up to sample n due to missing data

### Associations of sociodemographic characteristics and disease related factors with treatment adherence

Univariable linear regression analyses showed that mental health disorders, neoplasms and multimorbidity were negatively associated with the tendency to adhere to recommendation given by medical specialists. Cardiovascular diseases, diseases of the nervous system and currently working were positively associated with tendency to adhere. However, multivariable regression analyses showed that only mental health disorders (B = -11.113, *p* < 0.05) remained significantly associated (Table 3).

**Table 3:** Univariable and multivariable linear regression analyses of sociodemographic and disease related factors with treatment adherence

	Medical treatment adherence (*n* = 348)				Occupational treatment adherence (*n* = 229)			
	Univariable regression		Multivariable regression		Univariable regression		Multivariable regression	
	B^a ^	95% CI	B^a^	95% CI	B^a^	95% CI	B^a^	95% CI
Female gender	0.740	-3.417 – 4.897			-0.789	-6.568—-4.989		
Age	-0.059	-0.287—0.169			0.311**	0.010—0.612	0.245*	-0.078—0.569
Educational level								
Low (ref)								
Medium	2.461	-2.199—7.120			2.381	-4.345—9.108	4.116	-2.736—10.968
High	1.730	-3.686—7.145			-4.905*	-12.313—2.503	-1.665	-9.473—6.144
Working	2.845*	-1.455—7.146	0.902	-3.390—5.194	0.997	-4.841—6.834		
ICD-10 Type of disease								
Musculoskeletal disease	-2.565	-6.745—1.616			-2.595	-8.766—3.577		
Mental health disorder	-11.942**	-16.379—-7.505	-11.113**	-16.009—-6.217	-8.061**	-14.645—-1.478	-6.737*	-13.595—0.122
Neoplasm	-4.286*	-9.955—1.382	-4.655*	-10.351—1.040	2.774	-5.122—10.671		
Disease of the nervous system	7.419**	1.787—13.051	4.427*	-1.268—10.122	5.269*	-2.696—13.234	2.819	-5.269—10.906
Cardiovascular disease	4.867*	-0.797—10.530	4.690*	-1.053—10.434	3.388	-5.247—12.022		
Multimorbidity	-3.581*	-7.716—0.554	-0.174	-4.661—4.312	0.609	-5.359—6.577		

^a^B, Unstandardized regression coefficient, *CI* confidence interval, * *P* < 0.20, ** *P* < 0.05

Additionally, univariable linear regression analyses showed a negative association for high educational level and mental health disorders, and a positive association with age and diseases of the nervous system with the tendency to adhere to advice given by occupational health physicians. These results did not remain significantly associated in the multivariable analyses (Table 3).

### Associations of coping strategies, illness perceptions and perceived health with treatment adherence

The mean scores of each (sub)scale of both treatment adherence groups are presented in Table 1 and seem comparable between both groups. Multivariable regression analyses show that having an active approach as a coping strategy was positively associated with the tendency to adhere to advice given by medical specialists (B = 1.181, *p* < 0.001) and occupational health physicians (B = 0.925, *p* < 0.05). With regards to illness perceptions, results show that helplessness was negatively associated with the tendency to adhere (respectively B = -1.042, *p* < 0.001 and B = -1.697, *p* < 0.001), whereas the subscales acceptance (respectively B = 1.352, *p* < 0.001 and B = 1.214, *p* < 0.01) and perceived benefits (respectively B = 0.946, *p* < 0.001 and B = 0.993, *p* < 0.05) were positively associated with the tendency to adhere for both advices given by medical specialists and occupational health physicians.

While higher perceived mental health, based on the SF-12 [15], was associated with a higher tendency to adhere to medical specialist advice (B = 0.556, *p* < 0.01), it was not associated with a higher tendency to adhere to occupational health physician advice (B = 0.471, *p* = n.s.). Conversely, higher perceived physical health was associated with a higher tendency to adhere only to occupational health physician advice (B = 0.407, *p* < 0.05) (Table 4).

**Table 4:** Univariable and multivariable associations of coping strategies, illness perceptions and perceived health with treatment adherence

	Medical treatment adherence (*n* = 348)				Occupational treatment adherence (*n* = 229)			
	Univariable regression		Multivariable regression^a^		Univariable regression		Multivariable regression^a^	
	B^b^	95% CI	B^b^	95% CI	B^b^	95% CI	B^b^	95% CI
Coping strategies ^c^								
Active approach	1.452***	0.987–1.917	1.181***	0.670–1.693	0.963**	0.240–1.686	0.925*	0.115–1.734
Avoidance / abide	-0.593*	-1.165- -0.022	-0.335	-0.903–0.232	-0.733	-1.545–0.079	-0.614	-1.494–0.266
Illness perceptions ^d^								
Helplessness	-1.306***	-1.810- -0.803	-1.042***	-1.566- -0.518	-1.359***	-2.067- -0.651	-1.697***	-2.487- -0.906
Acceptance	1.672***	1.186–2.157	1.352***	0.832–1.871	1.210**	0.496–1.924	1.214**	0.424–2.003
Perceived benefits	1.329***	0.853–1.805	0.946***	0.417–1.474	0.926*	0.227–1.625	0.993*	0.239–1.746
Perceived health ^e^								
Physical health	-0.081	-0.329–0.167	0.096	-0.181–0.373	0.169	-0.133–0.471	0.407*	0.046–0.769
Mental health	0.811***	0.497–1.125	0.556**	0.215–0.879	0.573*	0.117–1.028	0.471	-0.053–0.995

^a ^Multivariable regression, adjusted for age, gender, educational level, work status, multimorbidity and ICD-10 diseases

^b ^B, Unstandardized regression coefficient

^c^ Coping strategies measured with the Utrecht Coping List (UCL) [13],

^d ^Illness perceptions measured with the Illness Cognition Questionnaire (ICQ) [14, 15]

^e^ Perceived health measured with the 12-item Short-Form Health Survey (SF-12) [16]

*CI* Confidence Interval, *p < .05, **p ≤ .01, ***p ≤ .001

### Sensitivity analyses

Of the participants, 158 had completed the adherence questionnaire for both the medical and occupational health physicians. The majority (*n* = 93, 58.9%) had a high tendency to adhere to the advice from both health professionals. A minority (*n* = 24, 15.2%) had a low tendency to adhere to both advice. Furthermore, 20.9% (*n* = 33) had a high tendency to adhere to the advice from the medical specialists only and 5.1% (*n* = 8) had a high tendency to adhere to the occupational health physicians’ advice only.

The four different groups only differed significantly on the number of participants having a mental health disorder. Of the participants who had a low tendency to adhere, almost half (45.8%, *n* = 11) had mental health disorders, whereas of the participants with a high tendency to adhere to both health professionals 18.5% (*n* = 17) had a mental health disorder (Table 5).

**Table 5:** Sensitivity analyses regarding low and high adherence combined for medical and occupational treatment adherence, *n* = 158

	Both low adherence (*n* = 24)	High medical adherence, low occupational adherence (*n* = 33)	Low medical adherence, high occupational adherence (*n* = 8)	Both high adherence (*n* = 93)
	N (%) or mean (± SD)	N (%) or mean (± SD)	N (%) or mean (± SD)	N (%) or mean (± SD)
Female gender	15 (62.5%)	17 (51.5%)	6 (75.0%)	49 (52.7%)
Age in years	49.8 (± 9.7)	48.9 (± 9.6)	55.9 (± 5.1)	50.7 (± 9.0)
Educational level				
Low	10 (41.7%)	8 (24.2%)	5 (62.5%)	31 (33.3%)
Medium	10 (41.7%)	15 (45.5%)	2 (25.0%)	38 (40.9%)
High	4 (16.7%)	10 (30.3%)	1 (12.5%)	24 (25.8%)
Working	12 (50.0%)	15 (46.9%)	5 (62.5%)	48 (52.7%)
ICD-10 Type of disease				
Musculoskeletal disease	8 (33.3%)	13 (40.6%)	3 (42.9%)	32 (34.8%)
Mental health disorder	11 (45.8%)*	5 (15.6%)*	2 (28.6)*	17 (18.5%)*
Cardiovascular disease	4 (16.7%)	5 (15.6%)	1 (14.3%)	14 (15.2%)
Neoplasm	3 (12.5%)	5 (15.6%)	1 (14.3%)	19 (20.7%)
Disease of the nervous system	1 (4.2%)	9 (28.1%)	1 (14.3%)	16 (17.4%)
Multimorbidity	9 (37.5%)	14 (43.8%)	4 (57.1%)	38 (41.3%)

*N* number of participants, *SD* standard deviation, * *P* < 0.05

## Discussion

In this cross-sectional survey study we studied treatment adherence, and the association of sociodemographic characteristics, disease related factors, coping strategies, illness perceptions and perceived health with the tendency to adhere to advice from medical and occupational health physicians in long-term sick-listed workers applying for disability benefits. Our results showed that 63.3% to 76.4% of the participants reported they were able to do what the physician told them to do in the past three months. When studying the associations of sociodemographic characteristics and disease related factors, only having a mental health disorder turned out to be negatively associated with the tendency to adhere to advice given by medical specialists in multivariable analyses. Furthermore, an active coping strategy, acceptance of the diseases, and perceiving positive long-term consequences of the disease were associated with a higher treatment adherence, whereas helplessness (focusing on the negative consequences) was associated with a lower adherence to both medical and occupational advice. A higher perceived mental health was positively associated with medical adherence, and a higher perceived physical health was positively associated with occupational adherence.

In general, the treatment adherence scores were slightly lower for advice given by occupational health physicians than for advice given by medical specialists. Although 80% did not find it difficult to do what the physician told them to do, still about 35% was not able to do what the occupational physician told them to do, and around 50% found it difficult to follow the advice. Furthermore, the mean scores of our study on the MOS-MPA questionnaire were 67.0 and 69.8 and are at the lower end compared to studies including cancer, hypertensive, diabetes and heart disease patients, which ranged from 64.6 to 81.5 [18, 19].

Although previous studies found several associations of sociodemographic factors, such as age and gender, with non-adherence, these did not remain in our multivariable analyses [20]. When looking at disease related factors, we found that being diagnosed with a mental health disorder was associated with a low tendency adhere. Furthermore, higher perceived mental health was associated with a higher medical adherence. This is in line with previous studies on adherence in patients with mental health problems. Reviews on treatment adherence including patients with schizophrenia, depression or anxiety reported non-adherence up to 89% [21–24]. Additionally, a meta-analysis across 31 studies and 18,245 participants diagnosed with a physical health condition, such as asthma, coronary heart disease, or diabetes, showed a significant association between depression and medical adherence; the odds of a depressed patient being non-adherent are significantly increased compared to a non-depressed patient [25]. These results indicate even when mental health disorder is not the primary diagnosis, physicians should be aware of psychological conditions, such as a depression, with respect to adherence to treatment advice. On the other hand, our study showed that a higher perceived physical health was positively associated with occupational adherence. Occupational advices might also include workplace adjustments. Advices on these adjustments are usually more concrete, and also more effective, in case of physical health problems compared to mental health problems [26]. This possibly makes it easier to adhere to these advices when physical health problems are not too severe, and when not struggling with mental health problems.

In our study an active coping strategy, and the subscales acceptance of the disease(s), and perceiving positive long-term consequences of the disease of the illness perceptions questionnaire were associated with a higher tendency to adhere, whereas helplessness (focusing on the negative consequences) was associated with a lower tendency to adhere to the advice given by medical specialists and occupational health physicians. Previous studies have also found other psychological risk factors, such as poor insight, denial of the illness, and negative attitudes, in patients with mental health disorders have been associated with medication non-adherence [8, 27]. In studies concerning physical diseases, associations have been found among coping strategies, illness perceptions and adherence. For example, in patients with coronary heart diseases, hypertension and chronic obstructive pulmonary disease, medical adherence was predicted by both a strong perception of personal and treatment control. On the other hand negative emotional response and negative beliefs in illness consequences was associated with adherence to low-fat diets and drug treatment [28–30]. Furthermore, in patients with rheumatoid arthritis, a higher score on reassuring thoughts (subscale of the UCL) was associated with willing to participate in an exercise program [31]. Coping strategies are not only associated with patient studies assessing adherence to medication, but also with intervention studies among healthy individuals. For example, one study found that pre-action self-efficacy (optimistic beliefs to participate) and formulating more coping plans such as motivational self-instructions, emotional control and use of resources, were associated with program adherence in healthy women who had participated in a physical or mental health activity program [32]. This suggests that regardless of whether an individual has been diagnosed with a disease, good communication about the disease is important, and may result in positive illness perceptions among patients. Intervention studies on altering the patients perceptions about their disease, showed improved functional outcomes, medical symptoms, and also on RTW after a myocardial infarction [33, 34]. Therefore, better communicating what a diagnosis entails, in an optimistic and compassionate way, patients can make a better understanding of the consequences of the disease on their lives [35]. Which will lead to more proactive coping strategies, and will feel less helplessness, will be able to accept the disease sooner, and perceive more positive long-term consequences then when they don’t know what to expect [36]. On the other hand, a self-fulfilling prophecy might occur; an increase in these mentioned perceptions will lead to higher treatment adherence, which will lead to more positive treatment outcomes and a better quality of life [36, 37].

### Strengths and limitations

To the best of our knowledge, this is the first study on adherence of long-term sick-listed workers to treatment advice of medical specialists and occupational health physicians. A strength of our study is that our study sample was not restricted to one specific diagnosis, we included patients with a wide variety of diagnosis, both physical and mental health disorders, which is a good reflection of the long-term sick-listed workers population and gave us the opportunity to compare results between the different diagnosis groups. Additionally, we did not only look at the associations of sociodemographic and disease related factors, which usually are no subject to change, but also at the associations of psychological factors. The latter can be affected by the participant self, and by communication strategies of the physician, and can subsequently increase the outcome.

Our study also has some limitations. One limitation is the low response rate. In total 5,407 work disability benefit applicants were contacted, but only 12% agreed to participate. Based on these numbers, a selection bias might have occurred resulting in an underestimation of our results, which might affect the internal and external validity of our study [38, 39]. Additionally, data were collected by means of self-report in which questions were asked on the tendency to adhere and a selection of factors that might be associated with adherence. It is known that self-report is subjective and may be susceptible to social desirability bias, we do not have data on the objective adherence to the advice or on non-adherence, nor is our data conclusive and adherence might be associations with many more factors than presented in our study. Finally, the cross-sectional design prevents us from drawing conclusions about causal relationships. Based on these limitations, our results are no solid evidence, and the associations should be interpreted carefully. However, the aim of our study was to explore possible associations with treatment adherence in long-term sick-listed workers applying for disability benefits. We believe our results do provide a relevant insight and can be built upon by future studies.

### Recommendations for further research and practice

Although various interventions were developed to increase RTW in short- and long-term sick-listed workers, these do not seem effective [6]. It is known that adherence has a great influence on the effectiveness of treatment in general, and also on days of sick leave and short-term disability [9]. However, the effect of adherence on long-term sick leave and RTW has not been studied yet. To gain more insight in the effect of adherence on RTW, adherence rates of our study should be compared to sick-listed workers who do RTW within 88 weeks of sick leave. Additionally, to increase RTW, interventions should be developed with an additional focus on active coping strategies and a positive illness perceptions, to increase adherence to the treatment and advice. Physicians should especially be aware of the adherence of sick-listed workers with mental health disorders, but also on those who focus on the negative consequences of their (physical or mental health) disorder.

## Conclusion

The tendency to adhere to medical and occupational advice in long-term sick-listed workers is relatively low. In order to increase RTW in this population, medical and occupational health physicians should be aware that specific groups of long-term sick-listed workers are at risk not to adhere to their advice. Future studies on RTW should focus on how to increase treatment adherence in long-term sick-listed workers and the effect of treatment adherence on RTW.

## Data Availability

The data that support the findings of this study are available from the corresponding author on reasonable request.

## References

[1] Organisation for Economic Co-operation and Development. Sickness, Disability and Work: Breaking the Barriers: A Synthesis of Findings Across Oecd Countries. Paris: OECD; 2010.

[2] Jetha A, Chen C, Mustard C, Ibrahim S, Bielecky A, Beaton D (2017). Longitudinal examination of temporality in the association between chronic disease diagnosis and changes in work status and hours worked. Occup Environ Med.

[3] Golics CJ, Basra MK, Finlay AY, Salek S (2013). The impact of disease on family members: a critical aspect of medical care. J R Soc Med.

[4] Singley SG (2003). Barriers to Employment among Long-term Beneficiaries: A review of recent international evidence.

[5] Saunders SL, MacEachen E, Nedelec B (2015). Understanding and building upon effort to return to work for people with long-term disability and job loss. Work (Reading, Mass).

[6] Vogel N, Schandelmaier S, Zumbrunn T, Ebrahim S, de Boer WE, Busse JW (2017). Return-to-work coordination programmes for improving return to work in workers on sick leave. Cochrane Database Syst Rev.

[7] DiMatteo MR (2004). Variations in patients' adherence to medical recommendations: a quantitative review of 50 years of research. Med Care.

[8] Julius RJ, Novitsky MAJ, Dubin WR (2009). Medication adherence: a review of the literature and implications for clinical practice. J Psychiatr Pract.

[9] Carls GS, Roebuck MC, Brennan TA, Slezak JA, Matlin OS, Gibson TB (2012). Impact of medication adherence on absenteeism and short-term disability for five chronic diseases. J Occup Environ Med.

[10] Bandura A (1986). Social foundations of thought and action: A social cognitive theory.

[11] Hays RD, Kravitz RL, Mazel RM, Sherbourne CD, DiMatteo MR, Rogers WH (1994). The impact of patient adherence on health outcomes for patients with chronic disease in the Medical Outcomes Study. J Behav Med.

[12] Verweij TA, Oosterveld P, Hoogstraten J (1998). Compliance in dentistry: general adherence, specific adherence and perceived dental health. Commun Dent Oral Epidemiol.

[13] Schreurs PJGW, G. v.d.; Brosschot, J.F.; Tellegen, B.; Graus, G. M. H. Handleiding Utrechtse Coping Lijst UCL (herziene versie). Lisse: Swets & Zeitlinger; 1993.

[14] Evers AWM, Kraaimaat FW, van Lankveld W, Jongen PJH, Jacobs JWG, Bijlsma JWJ (2001). Beyond unfavorable thinking: the illness cognition questionnaire for chronic diseases. J Consult Clin Psychol.

[15] Lauwerier E, Crombez G, Van Damme S, Goubert L, Vogelaers D, Evers AWM (2010). The construct validity of the illness cognition questionnaire: the robustness of the three-factor structure across patients with chronic pain and chronic fatigue. Int J Behav Med.

[16] Ware JE, Kosinski M, Keller SDJMc (1996). A 12-Item Short-Form Health Survey: construction of scales and preliminary tests of reliability and validity. Med Care.

[17] Mickey RM, Greenland S (1989). The impact of confounder selection criteria on effect estimation. Am J Epidemiol.

[18] Sherbourne CD, Hays RD, Ordway L, DiMatteo MR, Kravitz RL (1992). Antecedents of adherence to medical recommendations: results from the Medical Outcomes Study. J Behav Med.

[19] DiMatteo M, Hays R, Sherbourne C (1992). Adherence to cancer regimens: implications for treating the older patient. Oncology (Williston Park).

[20] Cheen MHH, Tan YZ, Oh LF, Wee HL, Thumboo J (2019). Prevalence of and factors associated with primary medication non-adherence in chronic disease: A systematic review and meta-analysis. Int J Clin Pract.

[21] Barkhof E, Meijer CJ, de Sonneville LM, Linszen DH, de Haan L (2012). Interventions to improve adherence to antipsychotic medication in patients with schizophrenia–a review of the past decade. European psychiatry : the journal of the Association of European Psychiatrists.

[22] Lingam R, Scott J (2002). Treatment non-adherence in affective disorders. Acta Psychiatr Scand.

[23] DiMatteo MR, Lepper HS, Croghan TW (2000). Depression is a risk factor for noncompliance with medical treatment: meta-analysis of the effects of anxiety and depression on patient adherence. Arch Intern Med.

[24] Haddad PM, Brain C, Scott J (2014). Nonadherence with antipsychotic medication in schizophrenia: challenges and management strategies. Patient related outcome measures.

[25] Grenard JL, Munjas BA, Adams JL, Suttorp M, Maglione M, McGlynn EA (2011). Depression and medication adherence in the treatment of chronic diseases in the United States: a meta-analysis. J Gen Intern Med.

[26] van Vlisteren M, van Oostrom SH, de Vet HCW, Franche RL, Boot CRL, Anema JR (2015). Workplace internetions to prevent work disability in workers on sick leave. Cochrane Database Syst Rev.

[27] Greenhouse WJ, Meyer B, Johnson SL (2000). Coping and medication adherence in bipolar disorder. J Affect Disord.

[28] Mosleh SM, Almalik MM (2016). Illness perception and adherence to healthy behaviour in Jordanian coronary heart disease patients. Eur J Cardiovasc Nurs.

[29] Hsiao CY, Chang C, Chen CD (2012). An investigation on illness perception and adherence among hypertensive patients. Kaohsiung J Med Sci.

[30] Olszanecka-Glinianowicz M, Almgren-Rachtan A (2014). The adherence and illness perception of patients diagnosed with asthma or chronic obstructive pulmonary disease treated with polytherapy using new generation Cyclohaler. Postepy dermatologii i alergologii.

[31] Vervloesem N, van Gils N, Ovaere L, Westhovens R, van Assche D (2012). Are personal characteristics associated with exercise participation in patients with rheumatoid arthritis? A cross-sectional explorative survey. Musculoskeletal Care.

[32] Evers A, Klusmann V, Schwarzer R, Heuser I (2012). Adherence to physical and mental activity interventions: coping plans as a mediator and prior adherence as a moderator. Br J Health Psychol.

[33] Petrie KJ, Cameron LD, Ellis CJ, Buick D, Weinman J (2002). Changing illness perceptiona sfter myocardial infarction: an early intervention randomized controlled trial. Psychsom Med.

[34] Broadbant E, Ellis CJ, Thomas J, Gamble G, Petrie KJ (2009). Further development of an illness perception intervention for myocardial infarction patients: a randomized controlled trial. J Psychosom Res.

[35] Freeman-Hildreth Y, Aron D, Cola PA, Wang Y (2019). Coping with diabetes: provider attributes that influence type 2 diabetes adherence. PloS One.

[36] Tiemensma J, Gaab E, Voorhaar M, Asijee G, Kaptein AA (2016). Illness perceptions and coping determine quality of life in COPD patients. Int J Chron Obstruct Pulmon Dis.

[37] Ivarsson B, Hesselstrand R, Radegran G, Kjellstrom B (2019). Health-related quality of life, treatment adherence and psychosocial support in patients with pulmonary arterial hypertension or chronic thromboembolic pulmonary hypertension. Chron Respir Dis.

[38] Chueng KL, ten Klooster PM, Smit C, de Vries H, Pieterse ME (2017). The impact of non-response bias due to sampling in public health studies: a comparison of voluntary versus mandatory recruitment in a Dutch national survey on adolescent health. BMC Public Health.

[39] Grimes DA, Schulz KF (2002). Bias and causal associations in observational research. Lancet.

